# Supplementary material for: Characterization and prediction of non-melanoma skin cancer incidence in China: Joinpoint regression and age-period-cohort model
Source: PLoS One. 2025 Jun 17;20(6):e0319486. doi: 10.1371/journal.pone.0319486 (PMC12173395; doi:10.1371/journal.pone.0319486)
Supplement: Attachment — Submitted filename: Response_to_Reviewers_auresp_2.docx [file pone.0319486.s004.docx]

PLOS ONE

Dec 13, 2024

PONE-D-24-40899

Characterization and prediction of non-melanoma skin cancer incidence in China: Joinpoint regression and age-period-cohort model

Dear editor and reviewers,

We appreciate editor and reviewers very much for their positive and constructive comments and suggestions on our manuscript entitled “Characterization and prediction of non-melanoma skin cancer incidence in China: Joinpoint regression and age-period-cohort model” (Manuscript ID: PONE-D-24-40899). Those comments are all valuable and very helpful for revising and improving our paper, as well as the important guiding significance to our researches. Based on these comments and suggestions, we have made careful modification on the original manuscript.

On the separate pages, we provided our response to the comments and suggestions, point by point, and highlighted the changes in the marked copy of the revision. We hope that our revision will be approved by the experts and reviewed favorably.

Sincerely,

Juan Mei Cao, MD

Journal Requirements:

Please review your reference list to ensure that it is complete and correct. If you have cited papers that have been retracted, please include the rationale for doing so in the manuscript text, or remove these references and replace them with relevant current references. Any changes to the reference list should be mentioned in the rebuttal letter that accompanies your revised manuscript. If you need to cite a retracted article, indicate the article’s retracted status in the References list and also include a citation and full reference for the retraction notice.

**Response：**Thank you very much for reminding us. After checking, we did not find any retracted articles, but several articles were in Chinese, and they were quoted to explain the situation in China.

Additional Editor Comments:

Thanks for the revision! However, I have noticed a few small things:

- Line 50-53: In my opinion, this is not written correctly. In 'western countries', the increase in NMSCs is mainly due to beauty ideals and related changes in leisure time (use of sunbeds, outdoor activites) and holiday behaviour (e.g. beach holidays). I cannot assess this in detail for emerging countries, but increasing air pollution (at least with particulate matter) does indeed lead to a reduction in UV. There are (conversely) European studies that show an increase in UV due to air pollution control measures. This should be presented in a more nuanced way, with sources.

**Response：**Thank you very much for your professional advice. The reasons for the increase in the incidence of NMSC are really complex and multiple, and the relationship with pollutants is also complex, and we have modified it in lines 51-54: “In recent years, the incidence of NMSC has been on the rise globally, possibly due to changes in lifestyle, increased ultraviolet (UV) exposure, and an aging population. For example, UV light is considered to be a carcinogen, which can induce and promote the occurrence and development of cancer.”

- Line 83: Why did you delete "statistical analysis"? The structure made sense, didn't it? As it is now ("statistical analysis" in line 126) it is not quite right, because I understand "statistical analysis" as a generic term under which "joinpoint regression model" etc. can be summarised.

**Response：**Thank you very much for your advice. We agree with you very much. It has been revised.

- Figure 1: In the illustration, the lines/dots (and colours) are virtually indistinguishable. I would recommend changing them, e.g. by combining solid and dashed lines.

**Response：**Thank you very much for your question. We have modified it again and divided it into five figures, which is the same as the other two figures’ structures. We hope to get your satisfaction.
